# Supplementary material for: Divergent roles for complement components C3 and C4 in controlling Klebsiella pneumoniae gut colonization and systemic dissemination
Source: bioRxiv. 2025 Oct 11:2025.10.09.681492. Preprint. [Version 1] doi: 10.1101/2025.10.09.681492 (PMC12632406; doi:10.1101/2025.10.09.681492)
Supplement: Supplement 1 [file media-1.pdf]

Supplemental Figures (1-4)

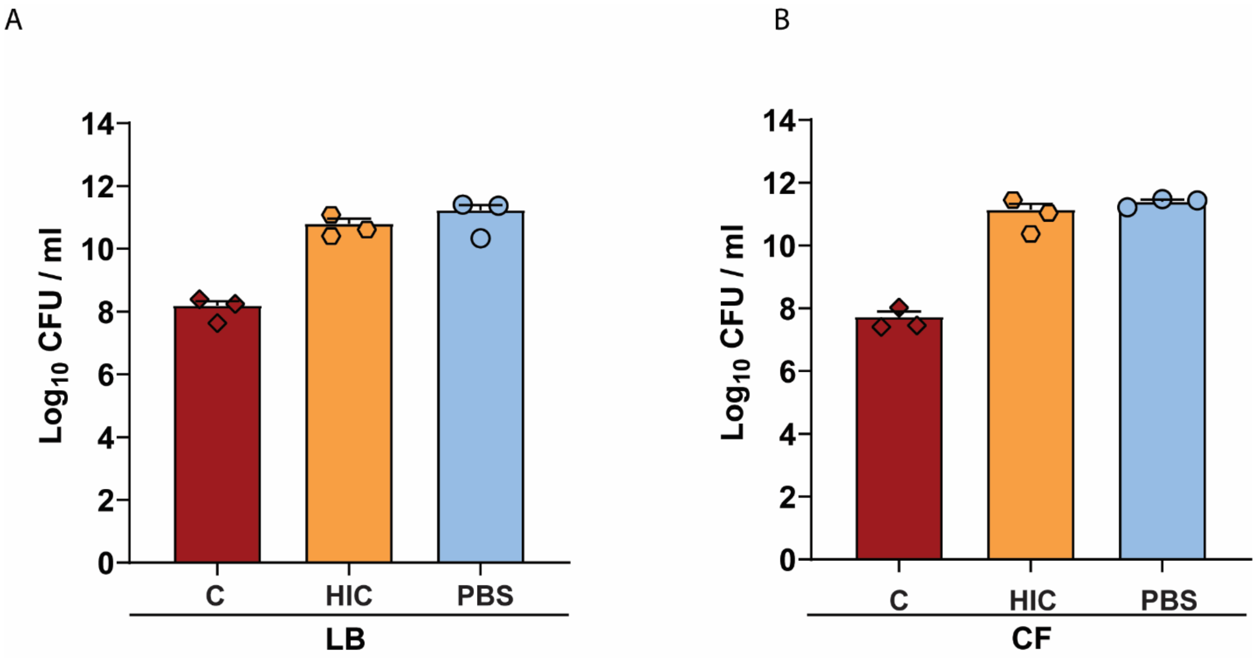

Figure S1. Capsule protects *Kpn* against complement-mediated killing (A–B) A *Kpn* isogenic capsule-deficient strain (*ΔwcaJ*) was incubated for 30 minutes at 37 °C in LB (A) or cecal filtrate (B) in presence of 10% rabbit complement, heat-inactivated complement, or PBS.

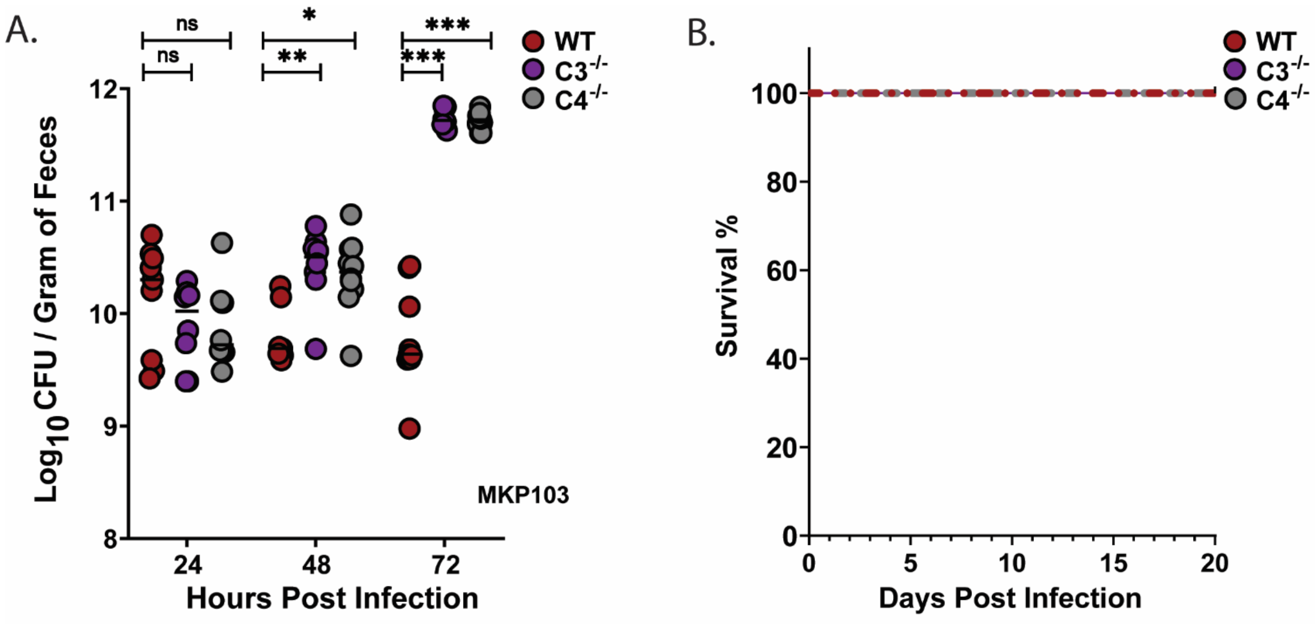

Figure S2. Bacterial burden of Kpn classical strain following antibiotic pretreatment. (A) Fecal shedding of mice colonized with MKP103 at 24-, 48-, and 72-hours post-gavage following ampicillin pretreatment (250 µg/mL in drinking water). Each data point represents an individual mouse; dotted lines denote the limit of detection (L.O.D.). All bacterial strains were grown overnight in LB, and mice were inoculated with ~10^7 CFU. Statistical significance was determined using the Kruskal–Wallis test with Dunn's post hoc test. n ≥ 9/group. *$P < 0.05$, **$P < 0.01$, ***$P < 0.001$, and ****$P < 0.0001$. (B) Kaplan–Meier survival curves of WT (red), C3$^{-/-}$ (purple), and C4$^{-/-}$ (gray) mice following MKP103 oral gavage.

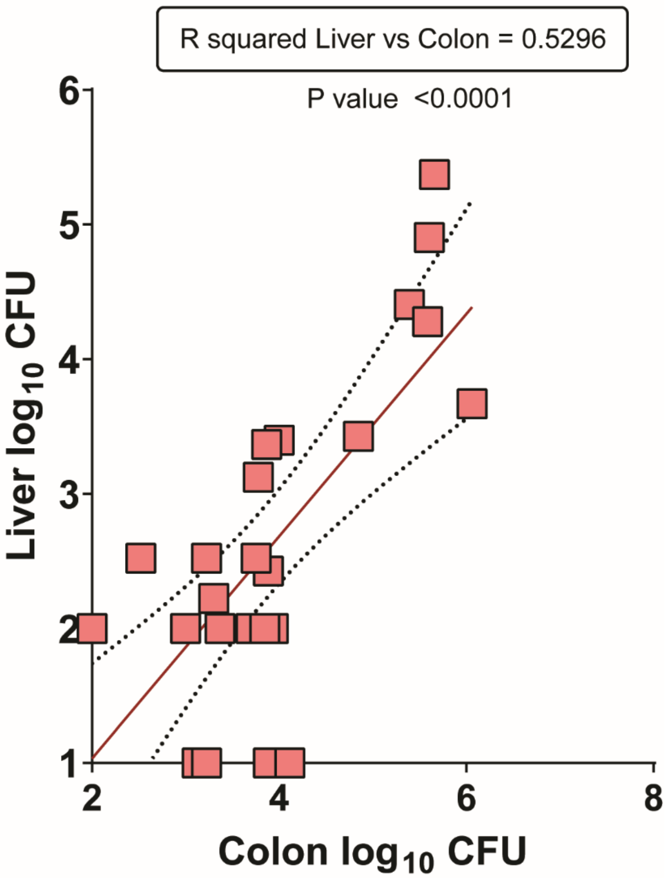

Figure S3. Elevated bacterial burden in the gastrointestinal tract correlates with increased bacterial dissemination to extraintestinal sites in wild type mice. (A) Correlation analyses of extra-intestinal organs (liver), bacterial burden of KPPR1S relative to gastrointestinal (colon) colonization at 24 hours PI in wild type mice, displayed as a simple linear regression curve. R squared for the lineal regression is listed, (n ≥ 27/group).

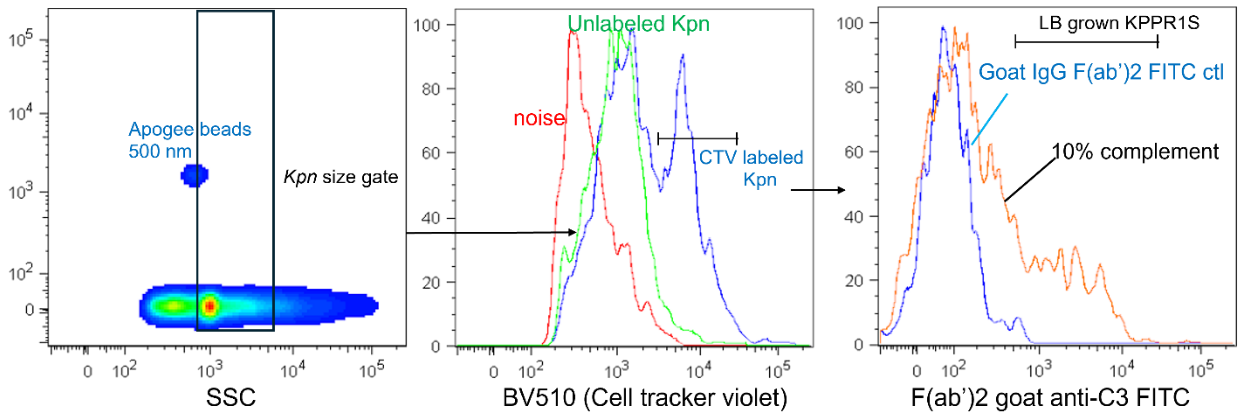

Figure S4. Flow cytometry gating strategy to quantitate C3b deposition on KPPR1S. *Kpn* was labeled with 1 uM Cell Tracker Violet for 30 minutes, washed with PBS containing 1% BSA, and incubated with 10% rabbit complement, 10% heat-killed complement, or PBS in RPMI at 37C for 1 hour. Bacteria was washed in PBS containing 1% BSA followed by staining with goat anti-C3/C3b F(ab')$_2$-FITC for 25 minutes. Bacteria were identified based on size using selective gating with Apogee beads followed by gating Cell tracker violet positive bacteria. Nonspecific goat IgG F(ab')$_2$-FITC was used to determine background staining. Mean fluorescent intensities (MFI) of C3b (FITC) signals were determined as a measure of C3b deposition level.
